# Biogenic synthesis of silver nanoparticles using *Rubus fruticosus* extract and their antibacterial efficacy against *Erwinia caratovora* and *Ralstonia solanacearum* phytopathogens

**DOI:** 10.1039/d3ra06723h

**Published:** 2024-02-14

**Authors:** Adnan Khan, Nisar Ahmad, Hina Fazal, Mohammad Ali, Fazal Akbar, Ishaq Khan, Mohammad Tayyab, Muhammad Nazir Uddin, Naveed Ahmad, Mostafa A. Abdel-Maksoud, Ibrahim A. Saleh, Naser Zomot, Hamada AbdElgawad, Kamran Rauf, Babar Iqbal, Marcelo Carvalho Minhoto Teixeira Filho, Mohamed A. El-Tayeb, Arshad Jalal

**Affiliations:** a Centre for Biotechnology and Microbiology, University of Swat Swat-19200 Pakistan adnankhan00119@gmail.com ahmadn@uswat.edu.pk alimoh@uswat.edu.pk fazalakbar@uswat.edu.pk ishaqqau@gmail.com nazir@uswat.edu.pk; b Pakistan Council of Scientific and Industrial Research (PCSIR) Laboratories Complex Peshawar 25120 Pakistan hina-fazalso@yahoo.com; c IBGE, The University of Agriculture, Peshawar Peshawar 25120 Pakistan muhammadtayyab@aup.edu.pk; d Botany and Microbiology Department, College of Science, King Saud University P.O. Box 2455 Riyadh 11451 Saudi Arabia Mabdmaksoud@ksu.edu.sa; e Faculty of Science, Zarqa University Zarqa 13110 Jordan isaleh@zu.edu.jo nzomot@zu.edu.jo; f Integrated Molecular Plant Physiology Research, Department of Biology, University of Antwerp 2020 Antwerp Belgium hamada.abdelgawad@uantwerpen.be; g Department of Horticulture, The University of Agriculture Peshawar Khyber Pakhtunkhwa 22620 Pakistan naveedhorticons@gmail.com raufkamran317@gmail.com; h School of Environment and Safety Engineering, Jiangsu University Zhenjiang 212000 China babar@ujs.edu.cn; i School of Engineering, Department of Plant Health, Soil and Rural Engineering, Sao Paulo State University Campus of Ilha Solteira 15385-000 Sao Paulo Brazil mcm.teixeira-filho@unesp.br arshad.jalal@unesp.br

## Abstract

In the current research, we produced green, cost-effective, eco-friendly silver nanoparticles using a single-step approach. Plants are considered highly desirable systems for nanoparticle synthesis because they possess a variety of secondary metabolites with significant reduction potential. In the current research, the dried leaf extract of *Rubus fruticosus* was utilized as a capping and reducing agent for the fabrication of silver nanoparticles, to prepare reliable biogenic silver nanoparticles and subsequently to investigate their potential against some common phytopathogens. The prepared silver nanoparticles were exploited to quantify the total flavonoid content (TFC), total phenolic content (TPC) and DPPH-based antioxidant activity. Different concentrations of aqueous extracts of plant leaves and silver nitrate (AgNO_3_) were reacted, and the color change of the reactant mixture confirmed the formation of *Rubus fruticosus* leaf-mediated silver nanoparticles (RFL-AgNPs). A series of characterization techniques such as UV-vis spectroscopy, transmission electron microscopy, energy dispersive X-ray analysis and X-ray diffraction revealed the successful synthesis of silver nanoparticles. The surface plasmon resonance peak appeared at 449 nm. XRD analysis demonstrated the crystalline nature, EDX confirmed the purity, and TEM demonstrated that the nanoparticles are mostly spherical in form. Furthermore, the biosynthesized nanoparticles were screened for *in vitro* antibacterial activity, antioxidant activity, and total phenolic and flavonoid content. The nanoparticles were used in different concentrations alone and in combination with plant extracts to inhibit *Erwinia caratovora* and *Ralstonia solanacearum*. In high-throughput assays used to inhibit these plant pathogens, the nanoparticles were highly toxic against bacterial pathogens. This study can be exploited for *planta* assays against phytopathogens utilizing the same formulations for nanoparticle synthesis and to develop potent antibacterial agents to combat plant diseases.

## Abstract 2

In the current research, we produced green, cost-effective, eco-friendly silver nanoparticles using a single-step approach.

## Graphical abstract

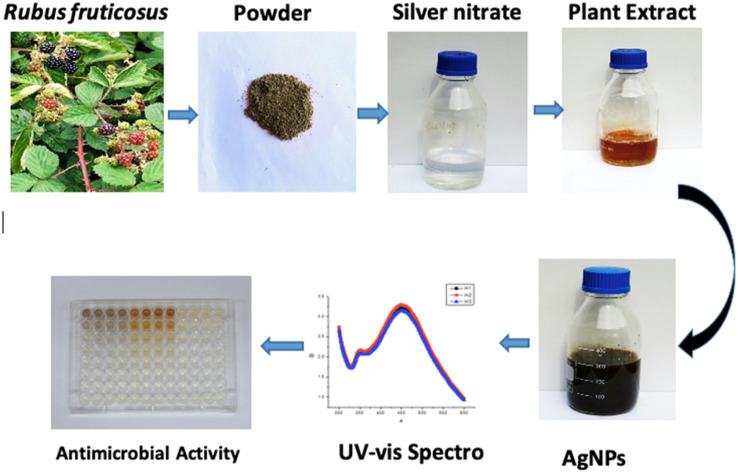

## 1 Introduction

Plant diseases cause massive agricultural losses all around the world. Traditional plant disease management techniques have harmed the environment as well as farmers' economic margins. An estimated 90% of pesticides are squandered away in the open field during spraying, which impacts the agricultural ecosystem and raises farmer expenses. Consequently, both the scientific community and the broader public have expressed apprehension regarding the ecological impacts stemming from excessive pesticide usage. Therefore, green nanostructures could be considered an effective and potential strategy to revolutionize agriculture by introducing new tools for disease management, rapid diagnostics, and higher nutritional uptake in plants.^1^

Nanotechnology has been one of the foremost fascinating scientific breakthroughs in recent years.^2^ In the fields of medicine and materials research, nanoparticles (NPs) have received international attention. Nanomaterials have a variety of applications, such as antibacterial, wound healing, and dental therapy applications, and are widely employed in biomedical devices across the world.^3^ NP-based treatments are said to be more effective due to their distinct optical and electrical features.^4^

Silver is a non-toxic, harmless, inorganic antibacterial substance that can kill up to 650 different microorganisms.^5^ Silver nanoparticles (AgNPs) are used in various fields such as medicine, food science, food packaging, agriculture, nano-sensors, drug delivery, cosmetics, textiles, and other industries as well.^6^ Various physiochemical characteristics of AgNPs reveal a variety of inhibitory mechanisms against microbes and therefore can be employed to manage several plant diseases in a safe manner.^7^ Plant-derived AgNPs have demonstrated promising biological potential. Plant-based silver nanoparticles of 5–100 nm size have been synthesized by several researchers.^8^

To date, a variety of physical and chemical approaches have been employed to develop silver nanoparticles. Both physical and chemical procedures for developing nanoparticles need a large amount of energy and produce toxic byproducts. This emphasizes the need to develop nanoparticle production techniques that are both sustainable and environmentally beneficial.^9^ The generation of nanoparticles using biological or phyto-mediated procedures is indeed an eco-friendly strategy.^10^ Eco-friendly substitutes include plant extracts, microbes, and enzymes.^11^ Green synthesis approaches involving plants and microbes have garnered attention because of their environmentally benign and biocompatible processes.^12^ Plant extracts are valuable in green synthesis, as they include a large proportion of bioactive components, such as alkaloids,^13^ tannins,^14^ flavonoids,^15^ and terpenoids,^16^ which can reduce silver ions to nanoparticles. The type of extract, reaction duration, pH, temperature, and silver nitrate solution directly/indirectly affect the size and shape of biogenic nanoparticles.^17^

Antibiotic resistance is a persistent problem that has pushed scientists to create new antimicrobial compounds. Antimicrobial resistance develops quickly, even before new drugs are introduced to the market. Antibacterial NPs, on the other hand, are expected to be able to minimize or prevent the formation of even more resistant strains by targeting many biomolecules simultaneously.^18^ Microbes are unlikely to acquire resistance to NPs because they have three modes of action for antimicrobial activity: oxidative stress, non-oxidative stress, and metal ion release.^19^ The usage of silver nanoparticles (AgNPs) in nanotechnology and nanoscience, particularly in nanomedicine, has increased recently.^20^

Antioxidant activity comprises inhibiting the onset of the oxidative chain reaction and limiting molecular oxidation to produce stable, nonreactive radicals.^21^ The antioxidant activity of biologically synthesized nanomaterials is influenced by the properties of the phytochemicals bonded to their surfaces.^22^ In plant extracts, the redox capacity of phytochemicals may play an important role in neutralizing free radicals and degrading peroxides.^23^ The antioxidant chemicals from the extract adhere to the surface of the nanomaterial, increasing their antioxidant capacity. The antioxidant activity of silver nanoparticles is higher than that of synthetic antioxidants.^24^

The genus *Rubus* belongs to the *Rosaceae* family, subfamily *Rosoideae*, and can be found all over the world. The shrub *Rubus fruticosus L.*, popularly known as blackberry, is thought to have originated in Armenia and is now found across Europe, Asia, South and North America, and Oceania.^25^ The stem and leaves of *Rubus fruticosus* include flavonoids, terpenes, glycosides, acids, and tannins, while the seed oil contains steroids, lipids, and vitamins with a wide spectrum of pharmacological effects. Plant extracts of blackberry are considered natural antioxidant, anticancer, antimicrobial, and anti-inflammatory agents.^26^

The primary objective of this research was to create biogenic silver nanoparticles and employ various characterization methods to distinguish high-quality silver nanoparticles. Additionally, we examined the antimicrobial properties of these AgNPs against plant pathogenic microorganisms to validate their potential applications. Moreover, we assessed the antioxidant capabilities of these nanoparticles. Our goal was to produce environmentally friendly and stable nanoparticles with substantial therapeutic value to address antibiotic-resistant bacterial strains.

## 2 Materials and methods

### 2.1 Plant collection

The *Rubus fruticosus* plants were acquired from the Swat District in Khyber Pakhtunkhwa. To remove dirt particles from the plant leaves, they were carefully cleaned with distilled water, dried, and ground with a blender or grinder to obtain a powder for extract preparation.

### 2.2 Extract preparation for silver nanoparticles

To prepare the extract for silver nanoparticles, 5 g of dried powdered leaves from *Rubus fruticosus* were combined with 1000 ml of distilled water in a conical flask. This mixture was then subjected to a 5 minutes boiling process. After boiling, the extract was double-filtered and stored at a temperature of 4 °C for further utilization.

### 2.3 Green synthesis of silver nanoparticles (AgNPs)

To produce silver nanoparticles, we combined plant extracts with silver nitrate solutions, as outlined in Table 1. We conducted a series of reactions to determine the most effective method for synthesizing AgNPs, involving various concentrations of both plant extract and AgNO_3_. These reactions utilized equal ratios of plant extract (ranging from 5 mg ml^−1^ to 1.25 mg ml^−1^) and AgNO_3_ solutions (also ranging from 5 mg ml^−1^ to 1.25 mg ml^−1^). After mixing 5 ml of each reactant in 15 ml Falcon tubes, the solutions were allowed to interact for 24 hours at room temperature. The alteration in color served as an indicator of Ag nanoparticle formation. The selection of the optimal biosynthesized nanoparticle was based on the evaluation of surface plasmon resonance and peak area.

**Table 1:** Biosynthesis of silver nanoparticles involved the utilization of different concentrations of both plant extract and silver nitrate

Silver nitrate (mM)	Plant extract (g L^−1^)		
	5.0 mM	2.5 mM	1.25 mM
10 mM	10 + 5	10 + 2.5	10 + 1.25
5.0 mM	5 + 5	5 + 2.5	5 + 1.25
2.5 mM	2.5 + 5	2.5 + 2.5	2.5 + 1.25
1.25 mM	1.25 + 5	1.25 + 2.5	1.25 + 1.25

### 2.4 Separation of silver nanoparticles

The reaction mixtures were loaded into Eppendorf tubes and centrifuged at 13 000 rpm for 15 minutes at room temperature. After centrifugation, the supernatant was discarded, and the pellet was reconstituted in deionized water. This entire centrifugation process was conducted in triplicate. Following each centrifugation round, the supernatant was removed, and the resulting pellets, which contained silver nanoparticles, were dried and stored in sealed containers.

### 2.5 Analysis of silver nanoparticles

#### 2.5.1 UV spectrophotometry

The formation of silver nanoparticles was investigated using UV-vis spectrophotometry. On a UV-vis spectrophotometer, the absorption spectra were examined at regular intervals between 300 and 600 nm.^27^

#### 2.5.2 X-ray diffraction

To assess the overall oxidation state and crystalline structure of the silver nanoparticles, X-ray diffraction (XRD) was employed. The nanoparticles were first purified by centrifugation and redispersion into deionized water, then freeze-dried and subsequently analyzed *via* XRD.

#### 2.5.3 TEM and EDX analysis

To assess the size, shape, and distribution of the silver nanoparticles, Transmission Electron Microscopy (TEM) was employed. A sample droplet was deposited onto a carbon-coated copper grid, ensuring even distribution. Following air-drying at room temperature, the grid's film was meticulously examined using a transmission electron microscope. Additionally, an elemental analysis of the biosynthesized silver nanoparticles was conducted, utilizing calibration with an energy-dispersive X-ray (EDX) detector, as detailed by Zahran *et al.*^28^

### 2.6 Antimicrobial evaluation

To examine the bactericidal effectiveness of AgNPs synthesized through green methods against phytopathogens (specifically, *Ralstonia solenecerum* and *Erwinia caratovora* subsp *Atroseptica*), a novel approach was adopted.^29^

First, the plant pathogens were cultivated in test tubes using nutrient broth as a growth medium. After a 24 hours incubation period, the optical density (OD) of the bacterial culture was assessed at a wavelength of 600 nm using UV spectrophotometry. Subsequently, different concentrations of nanoparticles, nanoparticles combined with plant extract, and plant extract alone were administered to the test microorganisms in 96-well plates, employing sterilized micropipettes to ensure precision. This procedure aimed to achieve optimal outcomes, and the microplate reader was employed for measurements. Control wells were also included, consisting of nutrient broth and bacteria but without nanoparticles. This entire process was repeated in triplicate. The 96-well plate was placed on a shaking incubator, and the OD was monitored at both 0 hours and 24 hours. After the 24 hours incubation period, the inhibition of bacterial species was observed. The percent activity of the nanomaterials was determined by eqn (1).

1 Activity (%) = Control − Treatment/Control × 100

### 2.7 In-planta antibacterial potential

The antibacterial efficacy of nanoparticles against *Erwinia caratovora* subsp *Atroseptica* in potato plants was evaluated, following the protocol of Alam *et al.*^30^ with some modifications. Potato plants in pots filled with sterilized soil were supplied with 10 ml of *Erwinia caratovora* subsp *Atroseptica* suspension (OD600:0.2). The suspension was inoculated around the tuber from whom the plant is grown. After three days, each pot was drenched with the appropriate volume (1000 μg mL^−1^) and (250 μg mL^−1^) of nanoparticles. The experiment was carried out in triplicate. Control was without nanoparticle treatment.

### 2.8 Assessment of antioxidant properties

We conducted a Free Radical Scavenging Assay (FRSA) following the protocol of Abbasi *et al.*^31^ to evaluate the antioxidant activity of each sample individually. Each cuvette was allocated 20 μl of the sample. Subsequently, 180 μl of the DPPH reagent was added to the cuvette, and the mixture was incubated in the absence of light for one hour. For the negative control, ideal concentrations of DPPH (180 μl) with DMSO (20 μl) and various concentrations of ascorbic acid (10, 05, 40, and 20 μg ml^−1^) were used. The absorbance at a wavelength of 517 nm was measured using a spectrophotometer. The FRSA activity was calculated by eqn (2).

2 % Scavenging DPPH Free Radical = 100 × (1 − AE/AD)

Here, AE represents the absorbance of the mixture at 517 nm in the presence of the sample, while AD signifies the absorbance of the DPPH solution without any additional substances.

### 2.9 Evaluation of phenolic and flavonoid contents

To assess the phenolic content (TPC), we followed the protocol outlined by Ahmad *et al.*^32^ During the total phenolic analysis, we gradually mixed 2.55 mL of distilled water with 0.1 mL of Folin Ciocalteus reagent and 0.03 mL of AgNPs. The resulting solution was then centrifuged for 15 minutes at 10 000 rpm, followed by a 30 minute incubation in the dark. To establish the standard curve, we used gallic acid (Sigma; 1.0–10 μg ml^−1^). The absorbance of both the sample and gallic acid was measured at 760 nm using a UV-visible spectrophotometer. The results were calculated as GAE (Gallic Acid Equivalent) in mg g^−1^ using eqn (3).

3 % Total Phenolic Content = 100 × (AS − AB)/(CF × DF)

Here, AS represents the absorbance of the sample, AB is the blank's absorbance, CF is the conversion factor derived from the standard curve, and DF represents the dilution factor.

For flavonoid analysis, we gently mixed 1.25 mL of distilled water with 0.25 mL of the extract, 0.5 mL of NaOH, and 0.075 mL of AlCl_3_ (5% w/v), following the method described by Ahmad *et al.*^32^ (2014). The resulting solution was then centrifuged at 10 000 rpm for 15 minutes and incubated in the dark for 30 minutes. The absorbance of each sample at 510 nm was determined using a UV-visible spectrophotometer. The total flavonoid content was calculated as RE (Rutin Equivalent) in mg g^−1^ dry weight of the sample using eqn (4).

4 % Total Flavonoid Content = 100 × (AS − AB)/(CF × DF)

Again, AS stands for the absorbance of the sample, AB is the blank's absorbance, CF is the conversion factor from the standard curve, and DF represents the dilution factor.

## 3 Results

**Fig. 1:**
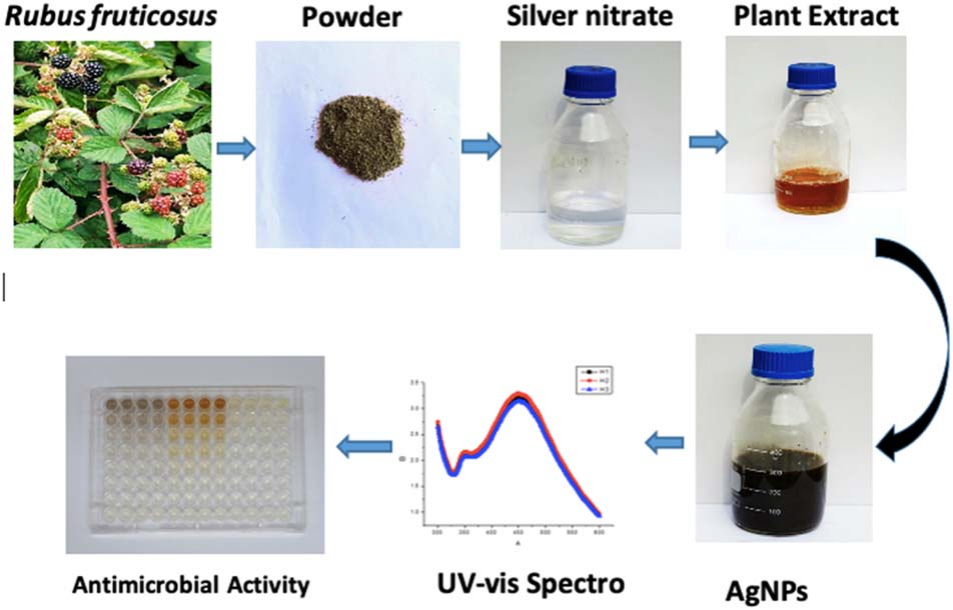
Pictorial presentation of the biogenic silver nanoparticles preparation from the leaves of *R. fruticosus* and its confirmation through UV-vis spectra and then its application for antibacterial activity.

In the current study, a green synthesis approach was used in which silver NPs were prepared. Leaves of *Rubus fruticosus* were used to prepare nanoparticles (AgNPs) of interest. As the procedure further proceeded, the antibacterial and antioxidant properties were also determined. The preparation of the required nanoparticles involved the use of silver nitrate at concentrations of 5.0 mg ml^−1^, 2.5 mg ml^−1^, and 1.25 mg ml^−1^, combined in equal proportions with plant volumes of 10 mM, 5 mM, 2.5 mM, and 1.25 mM. The overall pictorial overview of the experiment is given in Fig. 1.

### 3.1 Visual examination of the reaction mixture

**Fig. 2:**
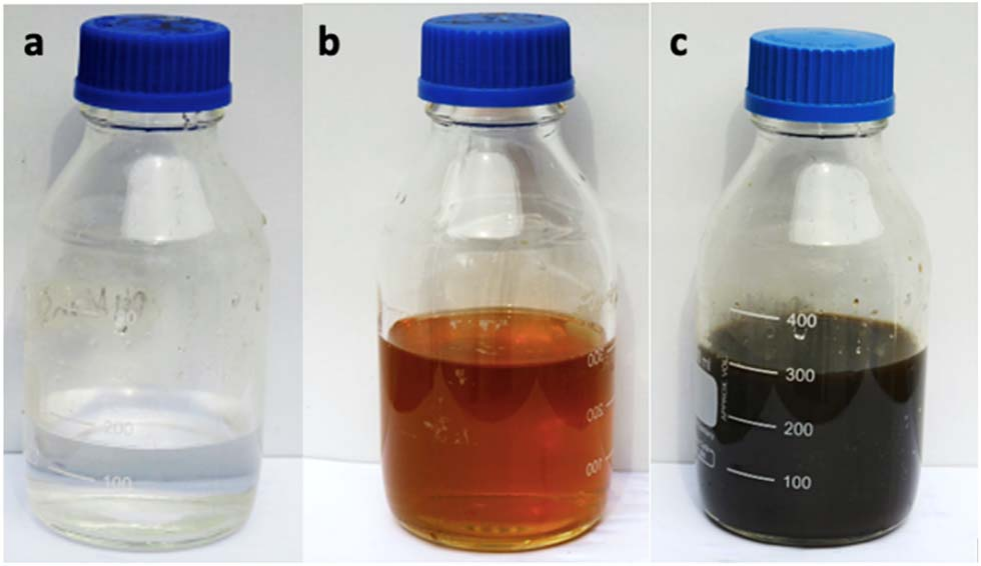
Alteration in the color of the reactant mixture (a) sliver nitrate (b) plant extract and (c) synthesis of nanoparticles.

The color of the mixture obtained from the extract of *Rubus fruticosus* changed to dark brown, which can be observed visually after twenty-four hours. This color change was consistent across different concentrations, which signifies the emergence of the surface plasmon resonance peak characteristic of nanoparticles synthesized through green methods. Fig. 2 visually represents this transition, displaying a light brown color of leaf extract (0 hours) that transformed into a rich dark brown shade after the 24 hours interval of mixing with silver nitrate solution.

### 3.2 Assessment of silver nanoparticles

#### 3.2.1 UV-visible spectroscopy

**Fig. 3:**
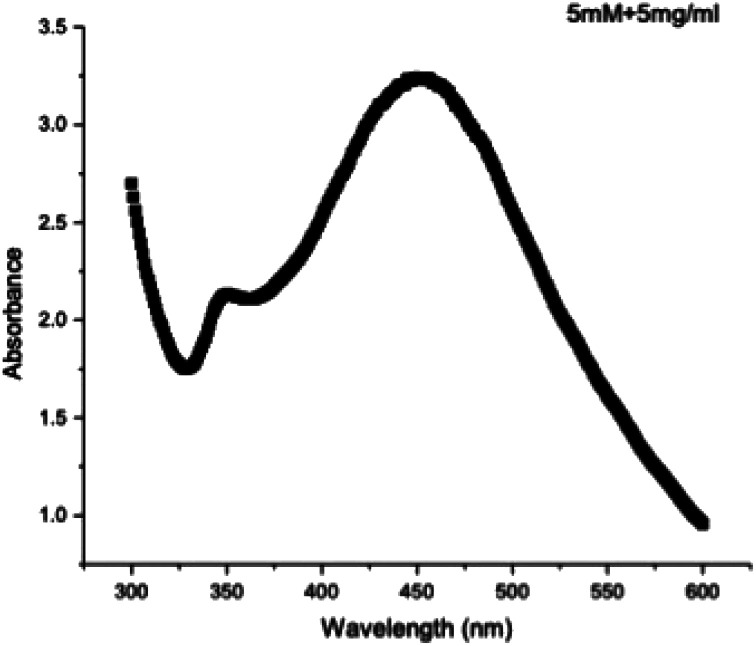
UV-visible spectroscopy was utilized to examine silver nanoparticles derived from the dried leaves of *Rubus fruticosus*.

The development of NPs can be determined by using UV-visible spectroscopy, which detects the reduction of silver ions from Ag^+^ to Ag within wavelengths of 300–600 nm. The Fig. 3 clearly shows that the highest peak was visible at 449 nm. This peak comes in the range of surface plasmon resonance for silver nanoparticles.^33^ It was determined that the optimum concentrations for the synthesis of AgNPs from *Rubus fruticosus* leaf extract were 5 mg ml^−1^ and 5 mM solution of silver nitrate.

#### 3.2.2 Energy dispersive X-ray analysis (EDX)

**Fig. 4:**
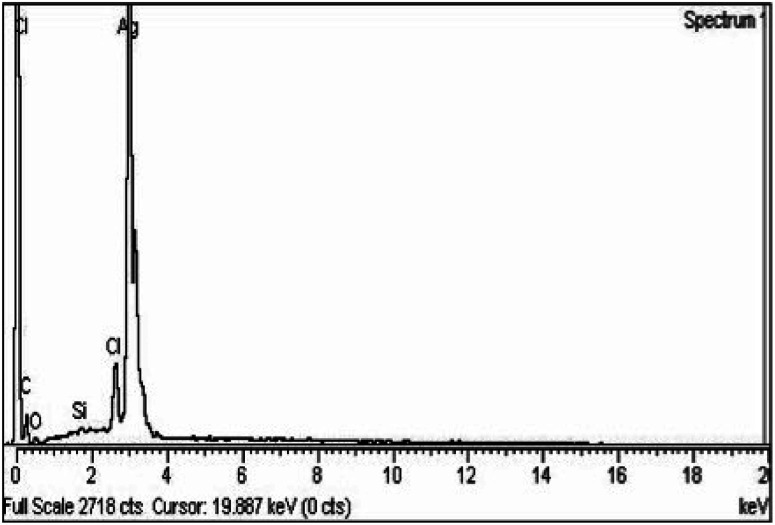
EDX spectra of RFL-silver nanoparticles prepared from the dried leaves of *Rubus fruticosus*.

It is essential to perform elemental analysis of synthesized nanoparticles using EDX spectra. The existence of silver NPs can be confirmed by proving the reduction of silver ions to elemental silver.^34^ The peak of AgNPs was observed at 3 keV along with some other elements (carbon, oxygen, silicon, and chloride). The reason for the peaks of C and O is their attachment to silver nanoparticles as a capping agent. Silicon and chloride peaks were displayed due to contamination from the grid where AgNPs were positioned. The results of EDX analysis showed that the proportion of silver was high (90%). Proportions of 3.59%, 3.52%, 1.94% and 0.37% were observed in the case of carbon (C), chloride (Cl), oxygen (O), and silicon (Si), respectively, as shown in Fig. 4.

#### 3.2.3 X-ray diffraction (XRD)

**Fig. 5:**
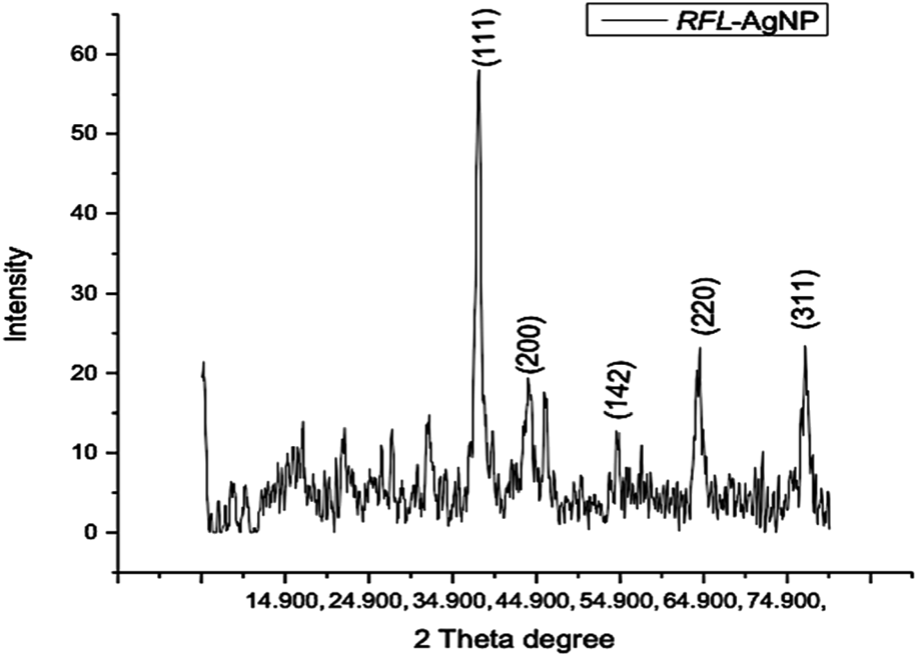
X-ray diffraction pattern of silver nanoparticles (AgNPs) synthesized from the dried leaves of *Rubus fruticosus*.

X-ray diffraction (XRD) analysis was employed to examine the structure and crystalline characteristics of the silver nanoparticles synthesized in this study. The XRD investigation of silver nanoparticles produced through green synthesis using *Rubus fruticosus* leaf extract exhibited distinct diffraction peaks at 38.1°, 44.4°, 54.5°, and 77.9°, as illustrated in Fig. 5.

The XRD spectrum provided clear evidence that the biosynthesized silver nanoparticles exist in a nanocrystalline form and possess crystalline attributes when compared to the standard. These peaks can be attributed to the (111), (200), (142), and (311) planes of silver crystals, respectively, as reported by Sathyavathi *et al.*^35^ Jemal *et al.*^36^ also observed similar results, supporting the characterization of silver nanoparticles as cubic, face-centered, and crystalline.

#### 3.2.4 Transmission electron microscopy (TEM)

**Fig. 6:**
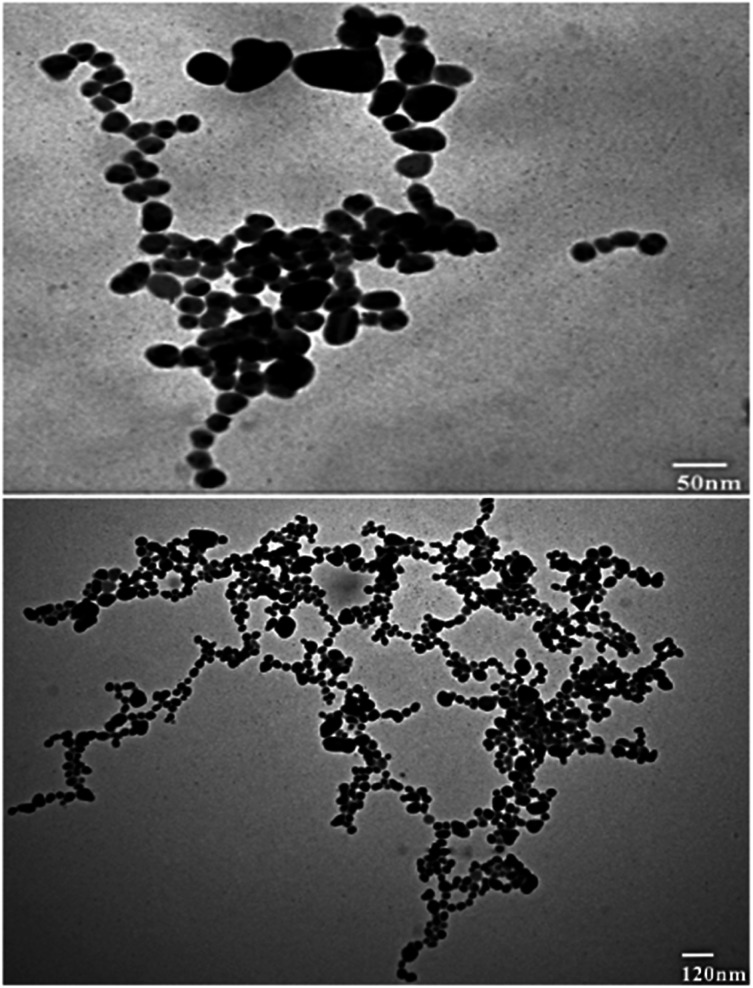
TEM micrograph of silver nanoparticles (AgNPs) synthesized from the dried leaves of *Rubus fruticosus*.

Transmission electron microscopy (TEM) was conducted to investigate the shape, size, and overall morphology of RFL-AgNPs. The TEM images vividly illustrate that the individual nanoparticles predominantly adopt a spherical form and tend to aggregate, lacking distinct morphological variations. This aggregation phenomenon is likely influenced by the presence of biological molecules within the leaf extract, which may have played a role in shaping these spherical RFL-AgNPs. As observed in the study, the aggregation could be attributed to the existence of secondary metabolites in the leaf extract (Jemal *et al.*,^36^ 2017). Additionally, the TEM images indicate that the size of the RFL-AgNPs falls within a range of 50 nm to 120 nm, as depicted in Fig. 6.

### 3.3 Assessment of antimicrobial potential

In order to determine the minimum inhibitory concentration (MIC) against *Ralstonia solanacearum* and *Erwinia caratovora*, a range of concentrations T1 (1000 μg mL^−1^), T2 (500 μg mL^−1^), T3 (250 μg mL^−1^), T4 (125 μg mL^−1^), T5 (62.5 μg mL^−1^), T6 (31.25 μg mL^−1^), T7 (15.62 μg mL^−1^), T8 (7.8 μg mL^−1^), T9 (3.9 μg mL^−1^), T10 (1.9 μg mL^−1^) of silver nanoparticles alone (NPs), nanoparticles embedded in plant extract (NPs + PE), and the filtrate of plant extract (PE) were utilized in triplicate to assess their effectiveness in inhibiting bacterial growth using eqn (1).

#### 3.3.1 Assessment of antibacterial effectiveness against *Ralstonia solanacearum*

**Fig. 7:**
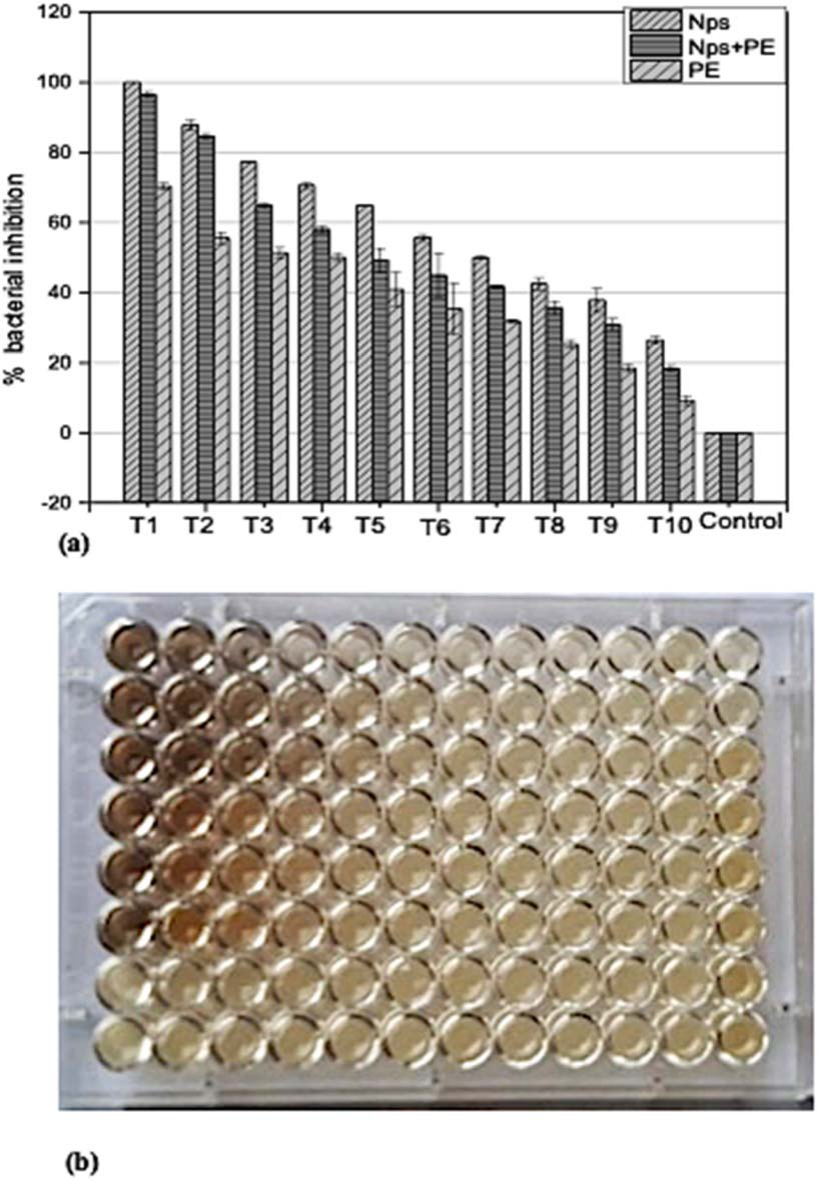
(a) Assessment of *Ralstonia solanacearum* inhibition using various concentrations (T1 = 1000 μg ml^−1^ to T10 = 1.9 μg ml^−1^) of nanoparticles NPs, nanoparticles combined with plant extracts (NPs + PE), and plant extract PE. (b) optical density (OD) plate of *Ralstonia Solanacearum* after 24 hours.

In the *In vitro* experiment, the highest concentration of T1 of nanoparticles NPs exhibited 100% inhibition of the test microbe. The same concentration of nanoparticles embedded in plant extract (NPs + PE) showed above 95% activity. At T2 the NPs and NPs + PE exhibited above 95% activity. At T3, T4 and T5 the NPs showed optimum activity of 60 to 80%, while the NPs + PE exhibited 40 to 60% bacterial inhibition for the same concentration. The plant extract (PE) at the highest concentration of T1 showed above 60% activity. At the lowest concentration the NPs, NPs + PE and PE exhibited less than 20% activity (Fig. 7). Notably, the inhibitory activity gradually decreased as the concentration decreased using equation-(1). These results suggest that both the nanoparticles (NPs alone) and the nanoparticles embedded in plant extract (NPs + PE) displayed significant efficacy against the phytopathogen *Ralstonia solanacearum*. Nevertheless, it's worth noting that NPs exhibited superior bacterial inhibition compared to the plant extract (PE) alone.

#### 3.3.2 Assessment of antibacterial effectiveness against *Erwinia caratovora*

**Fig. 8:**
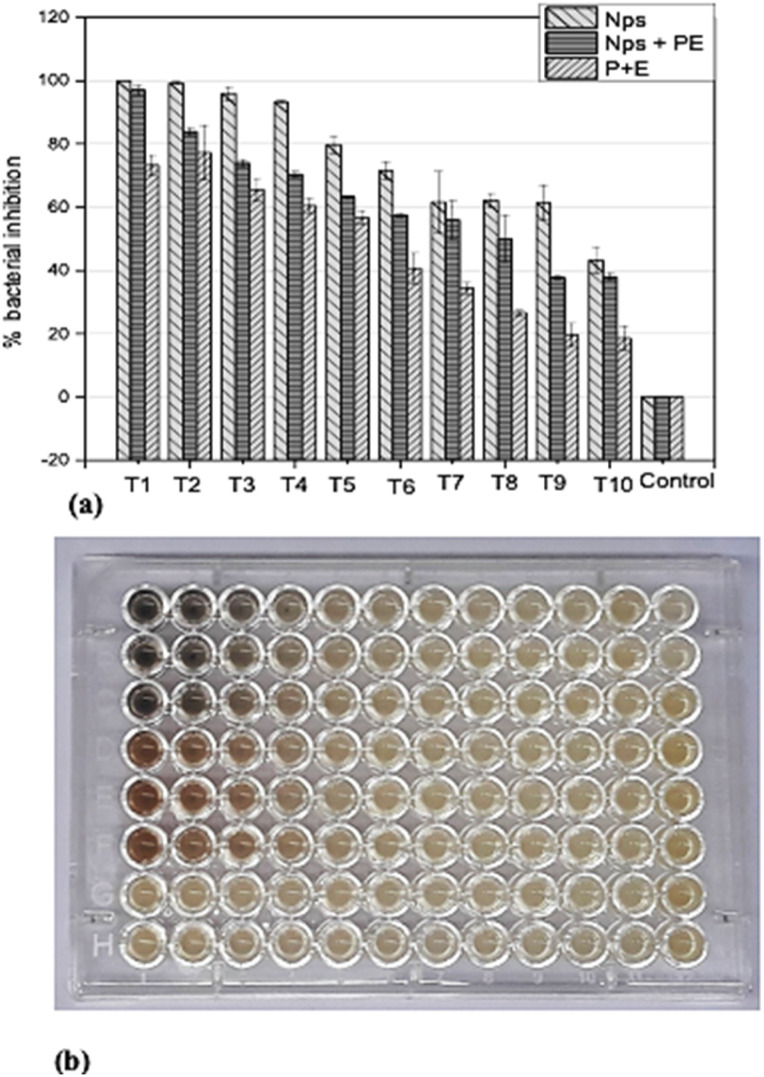
(a) Assessment of *Erwinia caratovora*. Inhibition using various concentrations (T1 = 1000 μg ml^−1^ to T10 = 1.9 μg ml^−1^) of nanoparticles NPs, nanoparticles combined with plant extracts (NPs + PE), and plant extract PE. (b) optical density (OD) plate of *Ralstonia Solanacearum* after 24 hours. (b) Microtiter plate image of *Erwinia caratovora* after 24 hours.

The highest concentration (T1) of NPs inhibited bacterial growth completely (Fig. 8a). At T2, T3 and T4 NPs exhibited above 90% bacterial growth inhibition, while the NPs + PE exhibited 70 to 90% activities for the same concentration. The PE at T1, T3 and T4 exhibited less than 80% activity. Unlike the dose-dependent bacterial inhibition the PE at T2 showed above 80% activity. At the lowest concentration, T10 the NPs and NPs + PE inhibited the test microbe by 40%, while at the same concentration, the PE inhibited the phytopathogen by less than 20% and these activities were determined using eqn (1).

### 3.4 Control of bacterial pathogenesis in potato plants

**Fig. 9:**
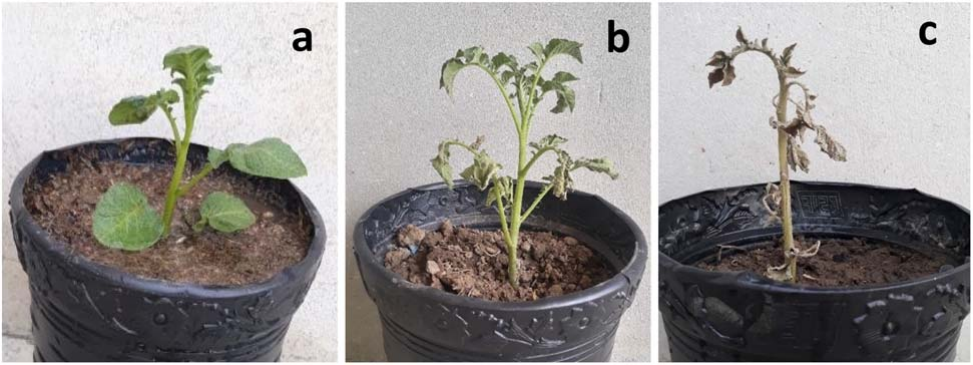
Controlling and protection of potato plants from phytopathogen (a) 250 μg mL^−1^ (b) NPs + PE and (c) control.

For this study, the potato plants were nurtured in a controlled environment. After post-inoculation, the control plant displayed severe symptoms and became dead. The plants treated with a lesser concentration of 250 μg mL^−1^ had no sign of illness. Furthermore, the plants were vigorous, and no obvious phytotoxicity was noticed. The plants were exposed to NPs alone and a combination of NPs + PE (Fig. 9)

### 3.5 Determination of total phenolic and flavonoid contents

**Fig. 10:**
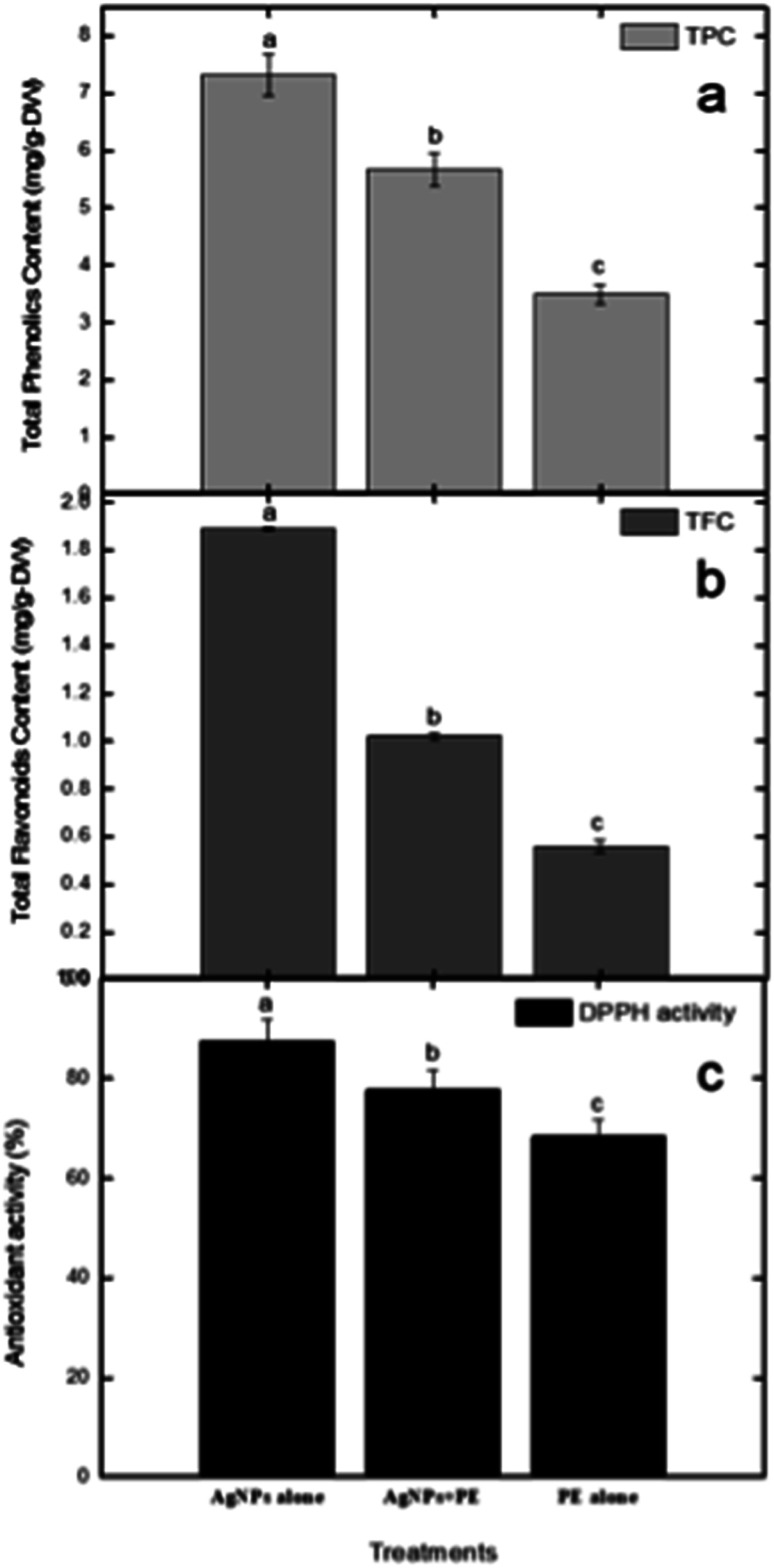
(a) The determination of total phenolic content, (b) the assessment of total flavonoids content, and (c) the evaluation of DPPH-based antioxidant activity conclusions.

In our current investigation, we assessed the total phenolic content using a method that involved three distinct samples: one containing RFL-mediated silver nanoparticles, another with only plant extract, and a third with a combination of nanoparticles and plant extract. The results revealed that RFL-AgNPs exhibited the highest total phenolic content (TPC) at 7.37 g GAE/100 g using eqn (3). Following closely were the uncentrifuged RFL-AgNPs, with a TPC of 5.67 g GAE/100 g, while the plant extract alone demonstrated a TPC of 3.49 g GAE/100 g (Fig. 10a and b). Moreover, in the determination of total flavonoid content, the RFL-AgNPs showcased a substantial content of 1.89 RE mg perg DW. In comparison, the uncentrifuged silver nanoparticles with plant extract and the plant extract alone exhibited total flavonoid contents of 1.02 RE mg per g DW and 0.56 RE mg per g DW, respectively.

### 3.6 Assessment of antioxidant capacity

In the DPPH assay, ascorbic acid was used as a standard to further determine the antioxidant properties of silver nanoparticles alone (NPs), nanoparticles embedded in plant extract (NPs + PE), and the filtrate of plant extract (PE). DPPH (red-colored and stable free radical having an absorption peak at 517 nm) interacts with some chemicals that are antioxidant in their characteristics and exhibit a reduction process (eqn (2)). It is now reduced so that its absorbance peaks decrease from 517 nm, giving a yellow color. Plant extracts show prominent antioxidant properties because of the presence of hydroxyl groups, confirming the presence of phenolic contents.^37^ To evaluate the DPPH-reducing potential of green-synthesized nanoparticles, we observed changes in color since the standard remained unchanged in color. The DPPH scavenging activity of AgNPs was notably significant compared to the standard.

Here, AE represents the absorbance of the mixture at 517 nm in the presence of the sample, while AD stands for the absorbance of the DPPH solution without any additives.

Results demonstrated that RFL-AgNPs exhibited strong DPPH scavenging activity at 87.33%, uncentrifuged RFL-AgNPs with plant extract displayed 77.67% DPPH scavenging activity (eqn (2)), and the plant extract alone showed 68.29% DPPH scavenging activity (as depicted in Fig. 10c). This heightened antioxidant activity in silver nanoparticles was attributed to the presence of functional groups acting as capping agents, their small size, and spherical morphology.^38^

## 4 Discussion

The realm of scientific research is currently experiencing rapid advancements in nanotechnology. Nanoparticles possess remarkable characteristics that set them apart from other materials.^32^ Scientists employ various methods, including physical, photochemical, chemical, biological, and electrochemical processes, to synthesize silver nanoparticles.^39^ Among these approaches, the use of plant extracts stands out as an excellent and cost-effective source for nanoparticle synthesis, thanks to its speed and minimal biological hazards compared to other methods.^40^ Ethanolic and aqueous extracts of plant leaves contain a rich array of chemicals, including alkaloids, flavonoids, phenols, tannins, carbohydrates, steroids, and saponins. These phytochemicals have been linked to various biological effects such as antihypertensive, anti-helminthic, diuretic, anti-diabetic, anti-malarial, antirheumatic, antiviral, anti-inflammatory, and anticancer properties in earlier studies.^41^

Numerous researchers have reported the biological production of AgNPs using plant leaves in the literature. Some examples include *Artemisia absinthium*,^27^*Crocus sativus*,^42^*Butea monosperma* bark extract,^43^*Nigella arvensis* leaf extract,^44^ as well as extracts from garlic, green tea, turmeric,^45^*geranium* leaves,^46^*Pisum sativum* outer peel,^47^*Passiflora edulis f. flavicarpa* leaves,^48^*Viburnum nervosum* leaves,^49^ T*amarindus indica* fruit shells,^50^*Phyllanthus emblica* fruits,^51^ pomegranate,^52^ and *Salvia officinalis* leaves.^53^

Recently, there has been significant interest in the sunlight-mediated green synthesis of silver nanoparticles due to its potential for harnessing clean solar energy. This approach offers simplicity, affordability, and the advantage of operating at room temperature. The process involves exposing a mixture of plant extract and silver nitrate to sunlight for a certain duration.^54^ Surprisingly, there are relatively few reports in the literature on the utilization of solar light for silver nanoparticle synthesis.

The literature is limited on the synthesis of AgNPs from the leaves of *Rubus fruticosus*, however, the synthesis of AgNPs with various biological activities prepared from different parts of other medicinal plants is widely reported.

In this study, silver nanoparticles were synthesized from a solution of silver nitrate and an aqueous extract of *Rubus fruticosus* leaves under sunlight, with a minimum reaction time of 10 minutes. Garibo *et al.*^55^ found that the conversion of silver ions to silver nanoparticles from *Lysiloma acapulcensis* took approximately 8 minutes. Some researchers attribute this rapid nanoparticle formation to the presence of abundant polar phyto-constituents in the aqueous leaf extract.^56^ Notably, the change in color of the reaction mixture from light brown to dark brown, observed visually, is indicative of this transformation. Similar color changes have been reported by Bhuyar *et al.*^57^ and Vidhu *et al.*^58^ This alteration in color is attributed to the surface plasmon resonance peak (SPR).^59^

The SPR peak was confirmed using a UV spectrophotometer, with an absorbance peak recorded at 449 nm for the absorption spectra of the green-synthesized silver nanoparticles. This result aligns closely with values of approximately 450 nm reported in previous studies.^60,61^ Typically, AgNPs exhibit a unique SPR ranging from 400 to 480 nm wavelengths,^62^ consistent with the findings of this research. Importantly, the SPR peak at 449 nm remained unchanged even after four months of incubation, indicating the stability and reliability of the green-synthesized RFL-AgNPs.

Four different XRD peaks, 38.1°, 44.4°, 54.5°, and 77.9°, at planes 111, 200, 142, and 311, respectively, were observed during the structural analysis of AgNPs prepared by *Rubus fruticosus*. This XRD analysis demonstrated that the synthesized silver nanoparticles exhibited a crystalline nature with a face-centered structure, in alignment with previous findings.^38,48^ Similar diffractions and peak patterns have been reported in earlier studies.^51,63^

TEM analysis was also studied for the synthesized nanoparticles, which indicated that silver nanoparticles were spherical in shape and uniform in structure and had a smooth surface, and a dark-colored coating was observed on the silver nanoparticles, indicating that phytochemicals from *Rubus fruticosus* act as capping agents and stabilization agents for silver nanoparticles.^64^ Synthesized nanoparticles were also studied using EDX analysis to study their elemental makeup, and a strong peak of 3 keV was recorded, indicating the presence of a higher proportion of silver in the nanoparticles which was 90%. Similar results were also observed in other literature studies.^49,61^

The antibacterial potential of silver nanoparticles revealed remarkable antibacterial activity against pathogenic bacteria. Different concentrations of silver nanoparticles were employed to assess their antibacterial efficacy against these pathogens. When studying the comparison of silver nanoparticles alone (NPs), and nanoparticles embedded in plant extract (NPs + PE), it was observed that NPs alone showed great inhibition against pathogenic bacteria. Similarly, the comparison of silver nanoparticles and *Rubus fruticosus* leaf extract was also studied against bacteria, and it was observed that NPs again showed a greater rate of inhibition compared to the RFL extract. These results were then compared with recent studies, and it was observed that silver nanoparticles proved to be the best against many pathogenic bacteria and have vast antibacterial activities.^65,66^ A recent study reported the antibacterial activity of silver nanoparticles prepared by using T*araxacum officinale* (dandelion) leaf extract.^8^

The antioxidant potential of synthesized AgNPs was also studied using the DPPH (2,2-diphenyl-1-picrylhydrazyl) assay by using uncentrifuged nanoparticles containing plant extract and by using RFL extract only. It was observed that AgNPs containing plant extract proved to have the best antioxidant potential compared to plant extract only. Various researchers have also studied the antioxidant activities of silver nanoparticles using different plant extracts.^8,38,48^

Moreover, nanotechnology plays a crucial role in various sectors due to their non-toxic, biodegradable, and compatible properties with few disadvantages, but these nano-objects are widely used as antioxidants, anti-fungal, anti-bacterial, and anti-cancerous and are dominant over other pharmaceuticals including *Rubus ellipticus* root derived NPs.^67–71^

In conclusion, it has been deduced from the current results that *Rubus fruticosus* leaves were exploited for the first time for the biogenic synthesis of silver nanoparticles that play a key role in inhibiting phytopathogens and improving the secondary metabolites. The current results are helpful in the formulation of natural bio-control agents with maximum antibacterial potential from this plant as compared to synthetic agents in controlling plant pathogens.

## Author contributions

Conceptualization: AK and NA; data curation and formal analysis: HF, AJ, and FA; investigation and methodology: MA, IK, MNU and KR; project administration and supervision: NA; resources: NA, HF and FA; software: AJ and BI; validation and visualization: AJ, MZU, IK, AK and NA; writing – original draft: NA and AK; writing – review & editing: AJ, HA, MCMTF; funding acquisition: MAAM, NZ and IAS.

## Conflicts of interest

There are no conflicts to declare.
