# Correction: Biogenic synthesis of silver nanoparticles using *Rubus fruticosus* extract and their antibacterial efficacy against *Erwinia caratovora* and *Ralstonia solanacearum* phytopathogens

**DOI:** 10.1039/d4ra90016b

**Published:** 2024-03-07

**Authors:** Adnan Khan, Nisar Ahmad, Hina Fazal, Mohammad Ali, Fazal Akbar, Ishaq Khan, Mohammad Tayyab, Muhammad Nazir Uddin, Naveed Ahmad, Mostafa A. Abdel-Maksoud, Ibrahim A. Saleh, Naser Zomot, Hamada AbdElgawad, Kamran Rauf, Babar Iqbal, Marcelo Carvalho Minhoto Teixeira Filho, Mohamed A. El-Tayeb, Arshad Jalal

**Affiliations:** a Centre for Biotechnology and Microbiology, University of Swat Swat-19200 Pakistan adnankhan00119@gmail.com ahmadn@uswat.edu.pk alimoh@uswat.edu.pk fazalakbar@uswat.edu.pk ishaqqau@gmail.com nazir@uswat.edu.pk; b Pakistan Council of Scientific and Industrial Research (PCSIR) Laboratories Complex Peshawar 25120 Pakistan hina-fazalso@yahoo.com; c IBGE, The University of Agriculture, Peshawar Peshawar 25120 Pakistan muhammadtayyab@aup.edu.pk; d Botany and Microbiology Department, College of Science, King Saud University P.O. Box 2455 Riyadh 11451 Saudi Arabia Mabdmaksoud@ksu.edu.sa; e Faculty of Science, Zarqa University Zarqa 13110 Jordan isaleh@zu.edu.jo nzomot@zu.edu.jo; f Integrated Molecular Plant Physiology Research, Department of Biology, University of Antwerp 2020 Antwerp Belgium hamada.abdelgawad@uantwerpen.be; g Department of Horticulture, The University of Agriculture Peshawar Khyber Pakhtunkhwa 22620 Pakistan naveedhorticons@gmail.com raufkamran317@gmail.com; h School of Environment and Safety Engineering, Jiangsu University Zhenjiang 212000 China babar@ujs.edu.cn; i School of Engineering, Department of Plant Health, Soil and Rural Engineering, Sao Paulo State University Campus of Ilha Solteira Sao Paulo 15385-000 Brazil mcm.teixeira-filho@unesp.br Arshad.jalal@unesp.br

## Abstract

Correction for ‘Biogenic synthesis of silver nanoparticles using *Rubus fruticosus* extract and their antibacterial efficacy against *Erwinia caratovora* and *Ralstonia solanacearum* phytopathogens’ by Adnan Khan *et al.*, *RSC Adv.*, 2024, **14**, 5754–5763. https://doi.org/10.1039/D3RA06723H.

The author regrets that the funding information was incorrectly shown in the acknowledgements section of the original manuscript. The corrected funding acknowledgement is as shown below.

The authors extend their appreciation to the Researchers Supporting Project number (RSPD2024R678) King Saud University, Riyadh, Saud Arabia.

The Royal Society of Chemistry apologises for these errors and any consequent inconvenience to authors and readers.

## Supplementary Material

